# From the Gut to the Bone: A Narrative Review of the Synovitis, Acne, Pustulosis, Hyperostosis, and Osteitis (SAPHO)-Inflammatory Bowel Disease (IBD) Overlap Syndrome

**DOI:** 10.7759/cureus.92230

**Published:** 2025-09-13

**Authors:** Abdulrahman A Almalaq, Abdulaziz Almasoud, Ebtissam Almeghaiseeb, Reem Alamro, Abdullah M Albishi, Seham Alrashidi, Abdulrahman Alrobayan

**Affiliations:** 1 Gastroenterology and Hepatology, Prince Sultan Military Medical City, Riyadh, SAU; 2 Gastroenterology, Prince Sultan Military Medical City, Riyadh, SAU; 3 Rheumatology, Prince Sultan Military Medical City, Riyadh, SAU

**Keywords:** autoinflammatory disorder, biologic therapies, crohn’s disease, diagnostic challenges, inflammatory bowel disease, musculoskeletal involvement, sapho syndrome, skin manifestations, therapeutic challenges, ulcerative colitis

## Abstract

Synovitis, acne, pustulosis, hyperostosis, and osteitis (SAPHO) syndrome is a rare autoinflammatory disorder primarily affecting the musculoskeletal system and skin. Its coexistence with inflammatory bowel disease (IBD) is uncommon and presents a unique diagnostic and therapeutic challenge, especially when musculoskeletal symptoms precede intestinal involvement. This review synthesizes current literature on the clinical, radiological, and therapeutic intersections between SAPHO syndrome and IBD to provide a comprehensive, multidisciplinary framework that supports early recognition and effective management.

A targeted narrative review of published case reports, clinical series, and relevant reviews was conducted using PubMed and Google Scholar, covering literature through 2024. In total, 14 publications describing SAPHO-IBD overlap cases were included in the synthesis, alongside additional literature informing pathophysiology and therapeutic context. Studies describing patients with concurrent SAPHO syndrome and Crohn’s disease or ulcerative colitis were included, with extracted data thematically organized to explore disease presentation, diagnostic delays, treatment strategies, and outcomes. The SAPHO-IBD overlap remains underrecognized and is frequently misdiagnosed as spondyloarthritis or infectious osteomyelitis. Diagnostic delays of more than one year were commonly reported across cases, most often due to misclassification. Imaging findings such as the “bull’s head” sign and sterile osteitis serve as diagnostic hallmarks but require a high index of clinical suspicion. Biologic therapies, particularly tumor necrosis factor-alpha (TNF-α) inhibitors and Janus kinase (JAK) inhibitors, have shown efficacy across both intestinal and osteoarticular domains; however, treatment challenges persist, including paradoxical dermatologic reactions and variable response across disease compartments. IBD-associated SAPHO syndrome represents a distinct but often overlooked clinical phenotype that challenges conventional diagnostic and therapeutic paradigms. Increased clinical awareness and interdisciplinary collaboration are essential to reduce diagnostic delays and facilitate individualized treatment approaches.

While limited by the heterogeneity of case-based evidence and the inherent constraints of narrative review methodology, this synthesis highlights consistent clinical and therapeutic patterns and underscores the need for standardized diagnostic criteria and systematic evaluation of long-term outcomes. To our knowledge, this review is the first to propose a unified clinical and immunopathologic framework specifically addressing SAPHO-IBD overlap.

## Introduction and background

SAPHO syndrome, an acronym for synovitis, acne, pustulosis, hyperostosis, and osteitis, is a rare, chronic autoinflammatory disorder characterized by sterile osteoarticular inflammation accompanied by distinctive cutaneous manifestations. It was first described as a unifying syndrome by Chamot et al. in the 1980s [1] and is now recognized as a heterogeneous entity that can mimic infectious, neoplastic, and autoimmune disorders. SAPHO shares overlapping features with chronic recurrent multifocal osteomyelitis (CRMO), a pediatric autoinflammatory bone disease characterized by sterile multifocal osteitis. While some authors consider CRMO part of the SAPHO spectrum, others emphasize distinctions based on age of onset, systemic associations, and frequency of cutaneous or gastrointestinal involvement [2]. CRMO typically manifests in children without cutaneous or gastrointestinal disease, whereas SAPHO more often presents in adults and may be associated with acne, pustulosis, or, in rare cases, inflammatory bowel disease (IBD). Although diagnostic criteria have been proposed, none are universally validated, and recognition remains challenging in clinical practice [3].

Although SAPHO syndrome primarily involves the osteoarticular and cutaneous systems, its coexistence with inflammatory bowel disease (IBD), including Crohn’s disease and ulcerative colitis, has been increasingly described in case reports and small series [4-7]. IBD is a chronic, immune-mediated disorder characterized by a broad spectrum of extraintestinal manifestations (EIMs), including peripheral arthritis, axial spondyloarthritis, and dermatologic conditions such as pyoderma gangrenosum and erythema nodosum [8,9]. The overlap of SAPHO syndrome and IBD constitutes a distinct but underrecognized phenotype in which gastrointestinal and osteoarticular symptoms may coexist, interact, or evolve asynchronously [10,11].

The SAPHO-IBD overlap presents a complex diagnostic and therapeutic challenge due to the asynchronous, multisystemic nature of its manifestations. Osteitis and hyperostosis are frequently mistaken for infectious osteomyelitis or degenerative joint disease, often leading to unnecessary antimicrobial therapy or orthopedic referral. Similarly, cutaneous features such as palmoplantar pustulosis and acneiform eruptions may be misdiagnosed as psoriasis, hidradenitis suppurativa, or adverse drug reactions [12]. Therapeutic decision-making is further complicated by the absence of standardized treatment algorithms and the risk of paradoxical immune-mediated reactions, particularly in patients treated with biologic agents [13].

The most widely used working criteria, proposed by Kahn, include combinations of sterile osteitis, hyperostosis, and characteristic skin lesions such as palmoplantar pustulosis or severe acne. However, these remain consensus-based rather than validated, and atypical presentations such as isolated bone pain or extra-axial involvement may fall outside their scope, increasing the risk of misclassification [4]. Diagnostic uncertainty is further compounded in cases with overlapping inflammatory bowel disease, necessitating multidisciplinary assessment and careful exclusion of other autoinflammatory or spondyloarthropathic syndromes.

This narrative review provides a comprehensive synthesis of current literature on SAPHO syndrome in the context of inflammatory bowel disease, with a focus on the clinical spectrum, shared pathophysiologic mechanisms, diagnostic pitfalls, and evolving therapeutic approaches. By integrating case-based evidence with interdisciplinary perspectives, this review aims to enhance clinical recognition of this underdiagnosed overlap and promote a more cohesive, multidisciplinary approach to its diagnosis and management.

## Review

Methodology

This narrative review was conducted with the aim of synthesizing current knowledge on the overlap between SAPHO syndrome and inflammatory bowel disease (IBD). We performed a structured literature search of PubMed, Embase, Scopus, and Web of Science databases from inception to July 2025. Search terms included combinations of “SAPHO syndrome,” “synovitis, acne, pustulosis, hyperostosis, osteitis,” “inflammatory bowel disease,” “Crohn’s disease,” “ulcerative colitis,” “spondyloarthritis,” “immunopathogenesis,” and “biologic therapy.”

No restrictions on study design were applied, and both clinical and basic science studies were considered. Articles were limited to the English language. Reference lists of key articles were manually screened to identify additional relevant publications. In total, 14 publications reporting SAPHO-IBD overlap cases were included in the synthesis, alongside additional literature informing pathophysiology, immunology, and therapeutic context. Publications were considered eligible if they addressed the epidemiology or prevalence of SAPHO in patients with IBD, described shared clinical, radiological, or immunologic features, discussed pathophysiologic mechanisms linking the two conditions, or reported therapeutic approaches and outcomes in patients with SAPHO-IBD overlap.

Given the narrative design, no formal risk-of-bias assessment or quantitative synthesis was performed. Instead, emphasis was placed on identifying key clinical concepts, recurring patterns, and gaps in the existing literature. The narrative design also carries inherent limitations, including potential publication bias, heterogeneity of case reports, and reliance on descriptive data. Importantly, most available case reports and series were published between 1992 and 2018, with few recent systematic datasets, underscoring both the rarity of SAPHO-IBD overlap and the lack of updated prevalence estimates. The review was organized thematically into sections on immunopathogenesis, clinical phenotypes, diagnostic challenges, and therapeutic considerations.

Epidemiology and published cases

SAPHO syndrome is a rare, chronic autoinflammatory disorder, with an estimated prevalence ranging from one in 10,000 to one in 100,000 individuals in the general population [1]. It typically presents between the third and fifth decades of life and shows a slight female predominance [2]. However, the true incidence is likely underestimated due to diagnostic underrecognition and clinical overlap with infectious osteitis, neoplastic processes, and seronegative spondyloarthropathies [3].

Although the coexistence of SAPHO syndrome and inflammatory bowel disease (IBD) is rare, it has been increasingly recognized in case reports and small clinical series. One of the earliest and most frequently cited retrospective studies, conducted by Naves et al., screened over 1,300 patients with IBD and identified three SAPHO cases, corresponding to a prevalence of 0.2% within the IBD cohort and 4.8% within their SAPHO registry [4]. In parallel, Colina et al. conducted a single-center cohort study of 71 SAPHO patients that provided important insights into the clinical and radiologic evolution of the disease [14], reinforcing the heterogeneity and diagnostic complexity of SAPHO in real-world cohorts. In addition to this cohort, several case reports have highlighted the clinical heterogeneity of SAPHO when associated with inflammatory bowel disease. Yamasaki et al. described a case of SAPHO complicated by pyoderma gangrenosum and IBD, initially masquerading as Behçet’s disease [15], while Siau and Laversuch reported SAPHO in ulcerative colitis responsive to pamidronate therapy [16]. More recently, Li et al. documented paradoxical cutaneous reactions induced by anti-TNF therapy in SAPHO, underscoring the therapeutic complexity of this overlap [17]. Subsequent reports have documented SAPHO in association with both Crohn’s disease and ulcerative colitis, with musculoskeletal symptoms frequently preceding gastrointestinal manifestations by several months or even years [5-7].

To date, no large-scale prospective studies have systematically assessed the prevalence of SAPHO syndrome among patients with IBD. Nonetheless, case-based evidence suggests that this overlap remains underrecognized, particularly in patients presenting with atypical chest wall pain, sterile osteitis, or nonspecific musculoskeletal symptoms [8]. Diagnostic delays are common and are frequently attributed to the insidious onset of disease and the misinterpretation of bone lesions as infectious, neoplastic, or degenerative in origin [9].

A summary of published pediatric and adult cases describing the co-occurrence of SAPHO syndrome and IBD is provided in Table 1. In total, 14 publications were identified, spanning 1992 to 2024, and together illustrate the heterogeneity of this overlap. Although older case series remain foundational, more recent systematic data are lacking, reflecting both the rarity of SAPHO-IBD overlap and the absence of prospective registries. These reports span both ulcerative colitis and Crohn’s disease across a wide age range and reveal a heterogeneous pattern of osteoarticular and cutaneous involvement. In several instances, treatment with biologic agents targeting shared inflammatory pathways such as tumor necrosis factor-alpha (TNF-α) inhibitors and Janus kinase (JAK)/STAT pathway blockers has resulted in concurrent remission of both intestinal and musculoskeletal manifestations. Cases labeled as chronic recurrent multifocal osteomyelitis (CRMO) were excluded to avoid diagnostic overlap.

**Table 1:** Summary of published SAPHO-IBD overlap cases Note: Cases labeled as chronic recurrent multifocal osteomyelitis are not included. IBD: inflammatory bowel disease, CD: Crohn’s disease, UC: ulcerative colitis, SAPHO: synovitis, acne, pustulosis, hyperostosis, and osteitis, PPP: palmoplantar pustulosis, HS: hidradenitis suppurativa, CT: computed tomography, MRI: magnetic resonance imaging, NA: not available or not applicable, IV: intravenous, TNF: tumor necrosis factor, IL: interleukin, ACW: anterior chest wall, NSAID: nonsteroidal anti-inflammatory drug

Author (year)	IBD type	Sequence of onset	SAPHO manifestations	Cutaneous findings	Imaging findings	Treatment	Outcome
Naves et al. (2013) [4]	CD and UC	IBD before SAPHO	ACW, spine	PPP, acneiform lesions	CT, MRI with osteitis	NSAIDs, anti-TNF (infliximab and adalimumab)	Partial improvement, variable response
Caporuscio et al. (2023) [5]	CD	IBD before SAPHO	ACW	Acneiform rash	MRI confirming osteitis	IL-12/23 inhibitor	Dual remission
Kim et al. (2024) [6]	UC	IBD before SAPHO	ACW osteitis	Not reported	MRI with active lesions	JAK inhibitors (tofacitinib)	Dual remission
Sayeed et al. (2019) [10]	UC	Concurrent	Sternoclavicular joint pain	PPP	CT with hyperostosis	NSAIDs, corticosteroids	Improved
Phillipps et al. (2024) [11]	UC	IBD before SAPHO	ACW, long bones	Not reported	MRI and bone scan	Bisphosphonates (zoledronic acid)	Complete remission
Yamasaki et al. (2003) [15]	CD	SAPHO before IBD	Multifocal osteitis	Pyoderma gangrenosum	Scintigraphy	Corticosteroids	Improved
Siau and Laversuch (2010) [16]	UC	IBD before SAPHO	ACW, clavicle	Not reported	CT scan	Bisphosphonates (pamidronate)	Improved
Marrani et al. (2018) [18]	CD and UC	SAPHO developed during IBD	Spine, pelvis	Acneiform eruptions	MRI	Anti-TNF (infliximab)	Controlled disease
Yang et al. (2024) [19]	UC	Concurrent	ACW	PPP	CT and MRI	NSAIDs, steroids	Resolved
Kang et al. (2018) [20]	CD	IBD before SAPHO	Sternoclavicular hyperostosis	Pyoderma gangrenosum	MRI	Corticosteroids, antibiotics	Resolved
Kotilainen et al. (1996) [21]	CD	IBD before SAPHO	Clavicular osteitis	Not reported	X-ray, CT	NSAIDs	Good response
Van Den Eynde et al. (2007) [22]	CD	Concurrent	ACW	HS	MRI	Anti-TNF (infliximab)	Mixed response
Dharancy et al. (1998) [23]	CD	IBD before SAPHO	ACW	PPP	Bone scan	NSAIDs	Improved
Kahn et al. (1992) [24]	CD and UC	Variable	ACW, spine, sacroiliac, peripheral	PPP, psoriasis, acne	X-ray, bone scan	Not specified	Not detailed

Pathophysiologic links between SAPHO syndrome and IBD

Cytokine-Mediated Pathways

**Figure 1:**
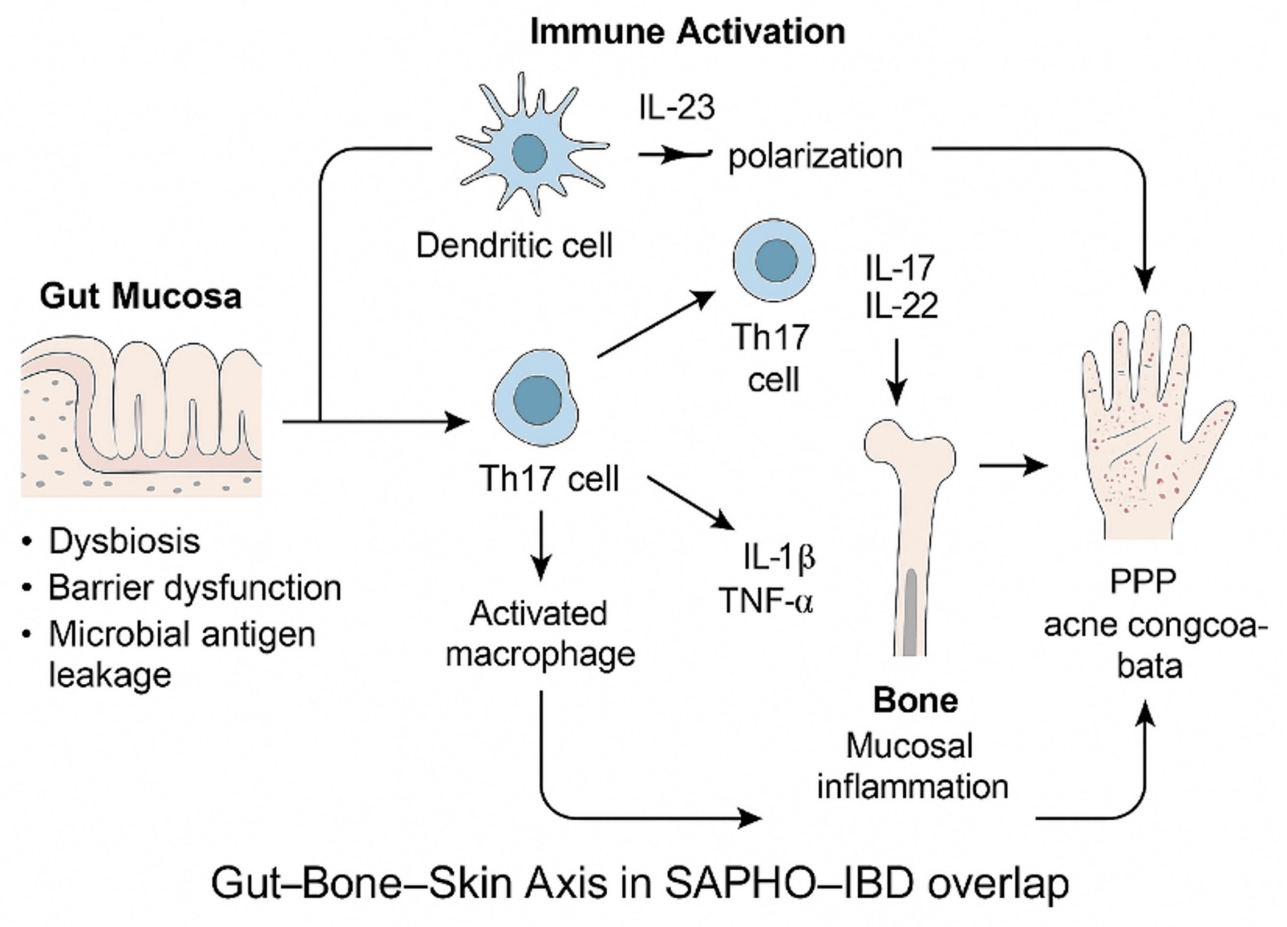
Conceptual diagram illustrating the gut-bone-skin axis in SAPHO-IBD overlap syndrome This figure demonstrates the shared immunopathogenic pathways linking intestinal inflammation in IBD with osteoarticular and cutaneous manifestations seen in SAPHO syndrome. Key inflammatory mediators, such as TNF-α, IL-1, IL-17, and IL-23, are involved in the activation of innate and adaptive immune responses across gut, bone, and skin tissues. Dysbiosis and microbial translocation in IBD may trigger systemic immune activation, promoting osteitis and pustular dermatoses. Conversely, bone marrow inflammation may serve as a reservoir for chronic systemic inflammation that exacerbates gut disease. This tri-directional axis highlights the clinical and therapeutic relevance of integrated care targeting shared cytokine pathways. SAPHO: synovitis, acne, pustulosis, hyperostosis, and osteitis, IBD: inflammatory bowel disease, IL: interleukin, TNF-α: tumor necrosis factor-alpha, IL-1: interleukin-1, IL-17: interleukin-17, IL-23: interleukin-23, Th17: T helper 17 (cell subtype), PPP: palmoplantar pustulosis Original figure created by the author

The coexistence of SAPHO syndrome and inflammatory bowel disease (IBD) highlights a shared immunopathogenic landscape rooted in innate immune dysregulation. Although originally considered a variant of seronegative spondyloarthropathy, accumulating evidence supports the reclassification of SAPHO as an autoinflammatory bone disorder driven primarily by aberrant neutrophil activation and the overexpression of proinflammatory cytokines (Figure 1) [25].

Both SAPHO and IBD are characterized by chronic, relapsing inflammation with considerable overlap in their immunologic underpinnings, particularly within the tumor necrosis factor-alpha (TNF-α) axis. TNF-α is consistently upregulated in both intestinal and osteoarticular inflammation, and the therapeutic success of inhibitors such as infliximab and adalimumab in managing manifestations of both diseases underscores the shared cytokine-driven pathogenesis. However, paradoxical worsening of cutaneous symptoms, including new or exacerbated pustulosis and psoriasiform eruptions, has been observed in some patients receiving anti-TNF therapy, suggesting a more nuanced and context-dependent cytokine network [26,27].

IL-23/IL-17 and JAK-STAT Pathways

In addition to TNF-α, the interleukin-23/interleukin-17 (IL-23/IL-17) axis plays a pivotal role in the pathogenesis of both intestinal inflammation and sterile osteitis. Elevated IL-17 and IL-23 levels have been demonstrated in patients with SAPHO, particularly during active bone inflammation, and targeted biologics such as ustekinumab, which inhibits the IL-12/23 p40 subunit, as well as IL-17 inhibitors, have shown therapeutic benefit in selected cases of SAPHO-IBD overlap [5-7,28].

Emerging evidence also implicates the Janus kinase (JAK)-signal transducer and activator of transcription (STAT) pathway in both conditions. This intracellular cascade mediates the effects of several cytokines, including IL-6, IL-12, IL-23, interferon-γ, and granulocyte-macrophage colony-stimulating factor (GM-CSF) [29-31]. Dysregulation of JAK-STAT signaling contributes to persistent osteoarticular inflammation in SAPHO and mucosal immune activation in IBD. Clinical efficacy of JAK inhibitors such as tofacitinib in ulcerative colitis, along with emerging use in refractory SAPHO cases where TNF-α inhibitors fail or worsen symptoms, further supports the concept of shared cytokine-driven immunopathogenesis [3,29,30].

Genetic Contributions

Genetic predisposition also appears to contribute to disease overlap. While HLA-B27 is not typically associated with SAPHO, other genetic variants implicated in autoinflammatory syndromes, including LPIN2 and NOD2 mutations, have been identified in both pediatric-onset SAPHO and familial IBD clusters [31,32]. These genetic abnormalities disrupt innate immune signaling, impair mucosal barrier function, and promote chronic inflammation across gastrointestinal and osteoarticular systems.

Microbial and Environmental Factors

Environmental and microbial factors provide another layer of convergence. In IBD, dysbiosis and altered intestinal microbial composition are well-established drivers of mucosal immune activation, while in SAPHO, microbial antigens, particularly *Cutibacterium acnes* from cutaneous or oral sources, are implicated in triggering sterile osteitis via mechanisms such as molecular mimicry and innate immune activation [9,12]. In individuals with compromised intestinal barrier function, these processes may be amplified, linking mucosal and osteoarticular inflammation in a biologically plausible manner.

Histologic and Clinical Parallels

Histologic and clinical parallels, including enthesitis and sterile osteitis, are also shared between SAPHO and IBD-associated spondyloarthropathy, further blurring the boundaries between these conditions [25]. Collectively, these findings suggest that SAPHO-IBD overlap exists along a unified immunologic spectrum rather than as two entirely distinct disease processes. Converging cytokine pathways, genetic predisposition, and environmental triggers such as microbial exposures appear to underlie this overlap, offering not only an explanation for its clinical coexistence but also a rationale for the use of targeted immunotherapies capable of addressing both conditions simultaneously [33].

Clinical presentations

The clinical presentation of SAPHO syndrome in the context of inflammatory bowel disease (IBD) is heterogeneous and often evolves asynchronously, which contributes to significant diagnostic delays. SAPHO encompasses a constellation of sterile osteoarticular manifestations such as osteitis, hyperostosis, and synovitis, along with diverse cutaneous lesions, and these features may precede, coincide with, or follow the onset of intestinal inflammation. This asynchronous pattern complicates clinical attribution and frequently delays comprehensive evaluation [34].

Musculoskeletal Manifestations

Musculoskeletal involvement is the most prominent domain, with sterile osteitis of the anterior chest wall representing the hallmark feature. The sternoclavicular, manubriosternal, and costochondral joints are most frequently affected, and in a 2020 Tunisian cohort, anterior chest wall disease was reported in nearly 90% of patients, often accompanied by synovitis and osteitis on magnetic resonance imaging (MRI) [33]. Clinically, such involvement may manifest as localized swelling, tenderness, or deep non-pleuritic chest pain, symptoms that are easily misattributed to infectious, neoplastic, or traumatic conditions [26]. Beyond the chest wall, the spine, particularly the thoracic vertebrae, can also be affected, occasionally presenting as spondylodiscitis or vertebral osteitis [14,26]. Less commonly, mandibular and temporomandibular joint disease occurs, sometimes mimicking osteomyelitis or malignancy [35]. Pelvic structures, including the sacroiliac joints, are another frequent site of involvement and are especially relevant in patients who have coexisting IBD and axial disease [34,36]. Importantly, musculoskeletal symptoms often precede the onset of gastrointestinal manifestations, highlighting the need to evaluate patients with unexplained sterile osteitis or atypical skeletal pain for possible underlying IBD [21,37].

Cutaneous Manifestations

Cutaneous features add another layer of complexity to the clinical picture. Palmoplantar pustulosis, severe acne variants, and hidradenitis suppurativa represent the most characteristic dermatologic associations of SAPHO in the context of IBD, although they may be subtle, misdiagnosed, or overlooked when they occur in isolation. Hidradenitis suppurativa, in particular, has gained recognition as a significant cutaneous hallmark and may coexist with palmoplantar pustulosis or acneiform eruptions [34]. Paradoxical psoriasiform eruptions further complicate the dermatologic profile; these have been reported in approximately 17% of patients with SAPHO treated with TNF-α inhibitors, often developing after 1-14 infusions. The underlying mechanism is thought to involve type I interferon activation mediated by plasmacytoid dendritic cells in response to TNF blockade, reinforcing the role of cytokine imbalance as a driver of paradoxical inflammation in this overlap population [17,38].

Gastrointestinal Manifestations

Gastrointestinal symptoms complete the clinical spectrum and include chronic diarrhea, hematochezia, abdominal pain, and unintended weight loss. Both ulcerative colitis and Crohn’s disease have been documented in association with SAPHO, and in many cases, the gastrointestinal diagnosis is delayed by months or even years relative to the onset of musculoskeletal disease [39,40]. This temporal disconnect obscures the recognition of an underlying systemic inflammatory process and often results in fragmented diagnostic pathways.

For this reason, a high index of suspicion for IBD is warranted in patients with SAPHO who develop gastrointestinal complaints, particularly in the presence of systemic inflammation or persistently elevated inflammatory markers [22,41].

Temporal and diagnostic challenges

Asynchronous Disease Evolution

The asynchronous evolution of musculoskeletal, dermatologic, and gastrointestinal manifestations in patients with SAPHO syndrome and inflammatory bowel disease (IBD) frequently leads to diagnostic delays and fragmented care, often confined within specialty-specific silos. Bone biopsies are commonly performed to exclude infection or malignancy, yet they typically reveal sterile inflammation consistent with SAPHO syndrome [14,42]. Early involvement of a multidisciplinary team, including rheumatology, gastroenterology, dermatology, and radiology, has therefore been emphasized as essential for improving diagnostic accuracy, minimizing unnecessary interventions, and expediting appropriate treatment [2,34-36].

Role of Imaging

Radiological evaluation occupies a central role in this process by helping distinguish sterile osteitis from infectious, neoplastic, or degenerative bone pathology, and in patients with IBD, early imaging can additionally detect subclinical musculoskeletal involvement and prompt timely referral [43,44].

The most distinctive radiological hallmark of SAPHO syndrome is hyperostosis with adjacent osteitis, particularly in the anterior chest wall, where sternoclavicular, manubriosternal, and costochondral joint lesions create a characteristic appearance across imaging modalities. Bone scintigraphy remains highly valuable in this context, with the so-called “bull’s head” sign (symmetric tracer uptake in the sternoclavicular joints and manubrium) considered pathognomonic for SAPHO and useful for identifying multifocal or clinically silent lesions in the spine and pelvis [1,25,44].

Computed tomography (CT) provides superior visualization of hyperostosis, joint erosions, and subchondral sclerosis, particularly within the anterior chest wall, axial skeleton, and mandible, and is especially effective in identifying early vertebral corner lesions that are often missed on plain radiographs [3,43,44]. Magnetic resonance imaging (MRI) offers the highest sensitivity for active disease by detecting bone marrow edema, synovitis, and enthesitis, which are frequently overlooked on other modalities [45]. It also assists in differentiating SAPHO-related lesions from infectious or malignant processes and is invaluable for identifying early or subtle disease [14,22].

Plain radiographs, although less sensitive, continue to serve as an accessible first-line tool in many patients with localized bone or joint pain. They may demonstrate osteosclerosis, periostitis, or joint space narrowing in advanced disease, but their limitations in identifying early lesions reduce their utility beyond initial evaluation [25,44]. In SAPHO-IBD overlap, MRI and bone scintigraphy are particularly useful, as they can delineate multifocal silent lesions and help distinguish SAPHO-related disease from IBD-associated sacroiliitis, especially when pelvic involvement is unilateral or lacks erosive features [10,44].

Histopathologic Evaluation

Histopathologic evaluation complements imaging in selected cases, especially when atypical or isolated skeletal lesions raise concerns for infection or malignancy. Biopsy typically demonstrates chronic sterile inflammation with a mixed lymphoplasmacytic infiltrate, osteitis, and marrow fibrosis, and cultures consistently fail to grow any pathogen despite early hypotheses implicating *Cutibacterium acnes* as a trigger [12,44,46].

While biopsy is unnecessary when the clinical and imaging findings are classic for SAPHO syndrome, it remains an important diagnostic step in elderly patients and in those at risk for malignancy, where differentiation is critical [44,47]. Several case reports have highlighted the value of radiological-pathological correlation in SAPHO-IBD overlap, describing concordant findings where CT and MRI demonstrated hyperostotic or edematous changes and histopathology confirmed sterile chronic inflammation. In one case of ulcerative colitis with first-rib osteitis, this correlation was key to securing the diagnosis and avoiding inappropriate antimicrobial or oncologic therapy [7,10,48-50].

Need for Integrated Diagnostic Approach

Such integration of imaging and biopsy underscores the need for a coordinated diagnostic approach, particularly in complex or high-risk patient populations.

Treatment strategies

The management of SAPHO syndrome in the setting of inflammatory bowel disease (IBD) presents significant clinical challenges because of the rarity of the overlap, the absence of standardized treatment guidelines, and the variability of therapeutic responses across skin, bone, and intestinal domains. Optimal care requires an individualized, symptom-targeted approach coordinated by a multidisciplinary team that includes gastroenterology, rheumatology, and dermatology specialists [8,51,52].

Conventional Therapies

Conventional therapies such as nonsteroidal anti-inflammatory drugs (NSAIDs), corticosteroids, and bisphosphonates have long been employed in SAPHO. NSAIDs are often regarded as first-line agents for osteoarticular manifestations, although their use in patients with concomitant IBD remains controversial due to the potential to exacerbate intestinal inflammation or trigger disease flares [8,14].

Corticosteroids can provide short-term relief for musculoskeletal and gastrointestinal symptoms, but their long-term use is limited by a lack of durable efficacy and the risk of adverse effects [14,51].

Bisphosphonates, including pamidronate and zoledronic acid, have demonstrated notable efficacy in refractory osteitis and hyperostosis, with case reports describing complete resolution of musculoskeletal symptoms after a single infusion of zoledronic acid in ulcerative colitis-associated SAPHO [11,53]. These benefits are thought to result from inhibition of osteoclast-mediated bone resorption and modulation of local inflammatory responses [35,54-56]. Despite encouraging evidence, bisphosphonates remain underutilized in the IBD population, likely reflecting limited awareness and their omission from standard therapeutic algorithms [54,57,58].

Biologic Therapies

The therapeutic landscape has expanded considerably with the introduction of biologic and targeted agents, although their effectiveness and safety vary.

Tumor necrosis factor-alpha (TNF-α) inhibitors such as infliximab and adalimumab have shown mixed results, with many patients experiencing significant improvement in intestinal and osteoarticular disease but others developing paradoxical skin reactions such as new-onset psoriasis or exacerbated hidradenitis suppurativa [59,60]. These adverse events often necessitate treatment discontinuation or switching to alternative biologics, underscoring the need for close dermatologic monitoring during anti-TNF therapy [61-63].

In this setting, therapeutic drug monitoring of infliximab has emerged as a valuable adjunct, with evidence that maintaining adequate serum drug levels correlates with improved outcomes. Strategies guided by drug levels, including dose optimization or switching agents, can therefore help achieve better disease control [64].

Beyond TNF blockade, interleukin-12/interleukin-23 inhibition with ustekinumab offers a promising option, particularly for patients who fail or are intolerant to TNF inhibitors. Ustekinumab, which targets the shared p40 subunit of IL-12 and IL-23, is already approved for Crohn’s disease and psoriasis, and, in the context of SAPHO-IBD overlap, has demonstrated benefit across gastrointestinal, cutaneous, and osteoarticular domains [65-67].

Targeted Small Molecules

Janus kinase (JAK) inhibitors provide another avenue of therapy. Tofacitinib, an oral JAK 1/3 inhibitor approved for moderate to severe ulcerative colitis, has been reported in case studies to induce remission of both intestinal and musculoskeletal manifestations in refractory patients or those with prior biologic failure [68,69]. Its oral administration and broad immunomodulatory effects make it particularly attractive for this rare overlap syndrome [51,67].

Experience with IL-17 and IL-1 inhibitors remains limited to isolated reports. Secukinumab, an IL-17 inhibitor, and anakinra, an IL-1 receptor antagonist, have been tried in refractory cases, but their role remains uncertain. IL-17 inhibition, in particular, is contraindicated in Crohn’s disease due to the risk of exacerbating intestinal inflammation, although cautious use may be considered in selected patients with ulcerative colitis without active luminal disease [70]. Given these risks, such agents should be reserved for carefully selected refractory cases with close clinical monitoring [32].

Ongoing Challenges

Despite therapeutic advances, the management of SAPHO-IBD overlap continues to be complicated by several challenges. Responses to therapy often differ across organ systems, with musculoskeletal, cutaneous, and gastrointestinal symptoms improving inconsistently to the same agent [14,71].

Furthermore, paradoxical inflammatory reactions, most notably psoriasiform and pustular flares during anti-TNF therapy, remain a significant clinical hurdle [60,63]. The absence of reliable predictive biomarkers compounds this problem, leaving clinicians reliant on trial-and-error approaches when selecting therapies [51].

These limitations highlight the importance of individualized treatment planning informed by multidisciplinary expertise. They also emphasize the need to expand clinical registries and real-world data collection to better define long-term outcomes and optimize therapeutic strategies [64,67].

Proposed Treatment Algorithm

Based on current case-based evidence and expert consensus, a stepwise approach to managing SAPHO-IBD overlap is recommended. For patients with mild musculoskeletal symptoms without active inflammatory bowel disease, a cautious trial of nonsteroidal anti-inflammatory drugs (NSAIDs) may be considered for localized symptom control, with or without the addition of bisphosphonates; however, the potential risk of IBD flare must be carefully monitored [14,72]. In cases of active IBD concurrent with SAPHO features, initiating targeted therapy with agents such as ustekinumab or tofacitinib can effectively address both intestinal and osteoarticular inflammation [65,66].

**Figure 2:**
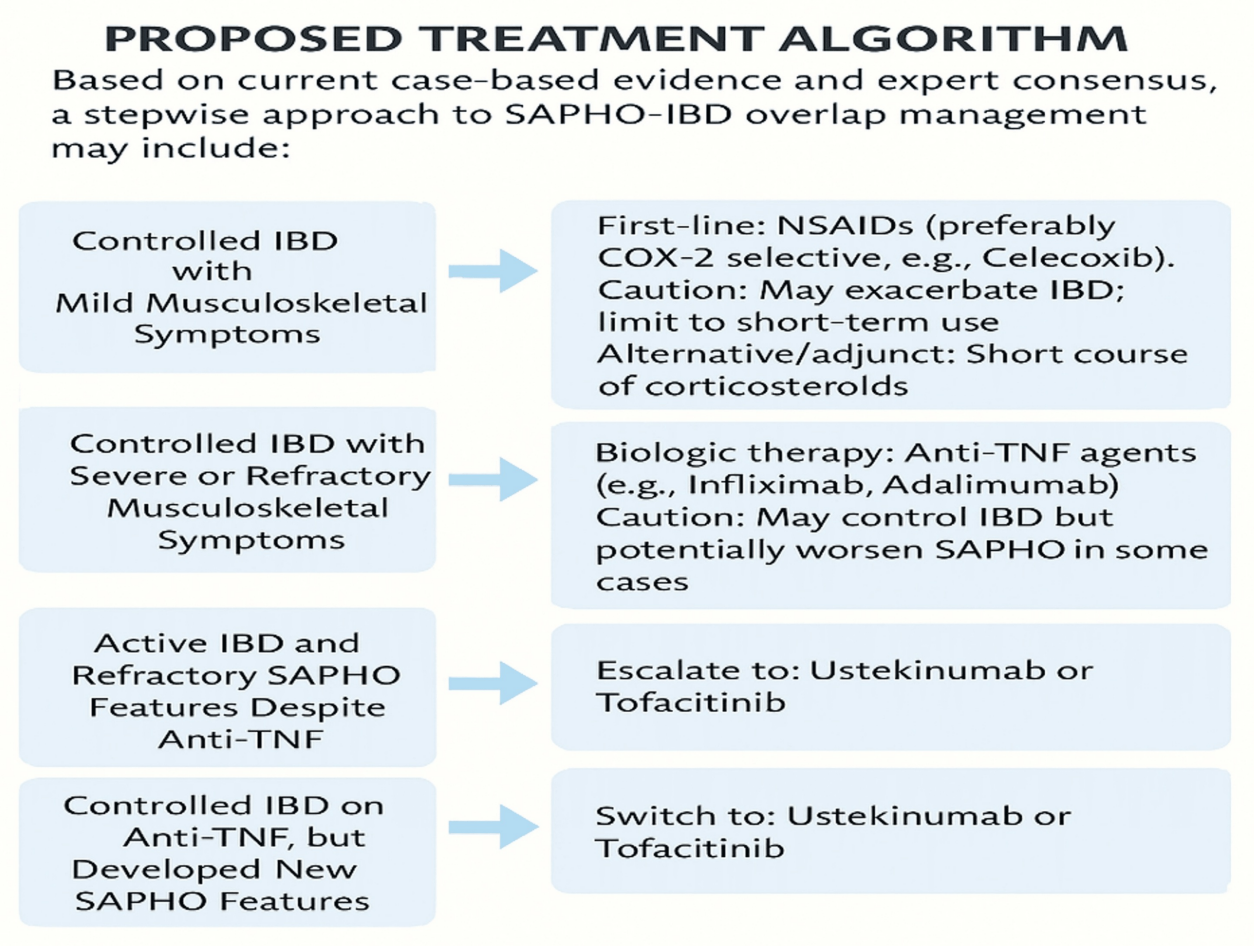
Stepwise approach to managing patients with overlapping SAPHO syndrome and IBD, based on disease activity and clinical presentation For patients with controlled IBD and mild musculoskeletal symptoms, short-term use of NSAIDs, preferably COX-2 selective agents such as celecoxib, is suggested, with caution due to potential IBD exacerbation. Corticosteroids may also be used briefly. Bisphosphonates such as zoledronic acid may be helpful in refractory musculoskeletal symptoms. In cases of active IBD with SAPHO features, anti-tumor necrosis factor (TNF) agents such as infliximab or adalimumab may address both gut and joint inflammation, although paradoxical SAPHO flares can occur. If refractory symptoms persist despite anti-TNF therapy, switching to IL-12/IL-23 inhibitors (e.g., ustekinumab) or JAK inhibitors (e.g., tofacitinib) may provide dual benefit. Similarly, patients with controlled IBD who develop new SAPHO symptoms on anti-TNF agents may benefit from transitioning to alternative biologics. SAPHO: synovitis, acne, pustulosis, hyperostosis, and osteitis, IBD: inflammatory bowel disease, TNF: tumor necrosis factor, COX-2: cyclooxygenase-2, NSAID: nonsteroidal anti-inflammatory drug, IL-12: interleukin-12, IL-23: interleukin-23, JAK: Janus kinase Original figure created by the author

For patients who demonstrate a favorable response to anti-TNF agents and maintain stable skin disease, continuation of therapy is advised alongside regular dermatologic monitoring to detect any paradoxical skin flares [22,61]. In refractory or paradoxical cases, switching to interleukin-12/interleukin-23 inhibitors or Janus kinase (JAK) inhibitors should be considered for those who exhibit poor response or adverse reactions to anti-TNF therapies (Figure 2) [62,67].

Diagnostic pitfalls and multidisciplinary care

Diagnosis of SAPHO syndrome in patients with inflammatory bowel disease (IBD) is frequently delayed because of its protean manifestations, overlap with more common conditions, and limited awareness of SAPHO-IBD overlap as a distinct clinical entity. Recognition therefore requires a high index of suspicion and coordinated evaluation across specialties [4,10,73].

One of the most frequent diagnostic pitfalls is misinterpreting anterior chest wall or mandibular pain as infectious osteomyelitis. Imaging in such cases may demonstrate lytic or sclerotic changes that resemble infection, often leading to unnecessary antimicrobial therapy or surgical intervention. Persistently negative cultures, lack of systemic infection, and poor response to antibiotics should instead prompt consideration of sterile osteitis, a hallmark of SAPHO syndrome [1,46].

Another challenge arises in distinguishing SAPHO from IBD-associated spondyloarthritis, as both may present with sacroiliitis and axial skeletal disease. However, SAPHO has a characteristic distribution (anterior chest wall, mandible, and metaphyseal regions of long bones) that is rarely seen in classic spondyloarthropathy, making recognition of this pattern critical to avoid misclassification [3,14].

Cutaneous lesions further complicate diagnosis. Palmoplantar pustulosis, acneiform eruptions, and hidradenitis suppurativa are sometimes misattributed to drug reactions, acne vulgaris, or psoriasis. Because these dermatologic findings often precede musculoskeletal symptoms, they represent important early diagnostic clues. Failure to recognize their systemic significance can lead to fragmented care confined to dermatology or infectious disease, rather than a unifying inflammatory diagnosis [3,58].

These pitfalls illustrate how the inherently multisystem nature of SAPHO-IBD overlap predisposes patients to compartmentalized evaluation. For example, a patient presenting with anterior chest wall swelling, chronic diarrhea, and pustular skin lesions may undergo separate consultations in orthopedics, gastroenterology, and dermatology without recognition of the overarching syndrome [4,10].

To provide practical guidance, we developed a comparative summary of distinguishing features between SAPHO syndrome, infectious osteomyelitis, and IBD-associated spondyloarthritis (Table 2). This quick reference framework highlights clinical distribution, microbiology, cutaneous manifestations, and imaging patterns that can aid clinicians in differentiating SAPHO from its common mimics.

**Table 2:** Key diagnostic differentiators between SAPHO and common mimics Note: Features listed are typical but not absolute; atypical presentations may occur. IBD: inflammatory bowel disease, SAPHO: synovitis, acne, pustulosis, hyperostosis, and osteitis

Feature	SAPHO syndrome	Infectious osteomyelitis	IBD-associated spondyloarthritis
Age of onset	Adults (20-50 years)	Any age, often children/young adults	Younger adults, peak in 20s-30s
Distribution	Anterior chest wall, mandible, spine, long bone metaphyses	Long bones (metaphysis/diaphysis), localized	Sacroiliac joints, axial skeleton
Cutaneous findings	Palmoplantar pustulosis, severe acne, hidradenitis suppurativa	Absent	Erythema nodosum, pyoderma gangrenosum
Systemic features	IBD association, sterile osteitis	Fever, elevated inflammatory markers, bacteremia	IBD association, peripheral arthritis
Microbiology	Sterile, cultures negative	Positive cultures (blood/bone)	Sterile
Response to antibiotics	Poor	Good (if pathogen targeted)	Not applicable
Imaging	Hyperostosis, “bull’s head” sign, multifocal sterile osteitis	Lytic lesions, abscess, periosteal reaction	Sacroiliitis, erosions, ankylosis

Because of these challenges, early multidisciplinary collaboration is essential. Involvement of gastroenterology, rheumatology, dermatology, radiology, infectious disease, and pathology enhances diagnostic accuracy, reduces unnecessary interventions, and facilitates individualized treatment planning [74,75]. Linking these specialties is particularly important in SAPHO-IBD overlap, where coordinated assessment can shorten diagnostic delays and directly address the common pitfalls outlined above (Table 3).

**Table 3:** Role of multidisciplinary evaluation in SAPHO-IBD overlap SAPHO: synovitis, acne, pustulosis, hyperostosis, and osteitis, IBD: inflammatory bowel disease, DMARD: disease-modifying antirheumatic drug, NSAID: nonsteroidal anti-inflammatory drug, CT: computed tomography, MRI: magnetic resonance imaging

Specialty	Primary role
Gastroenterology	Diagnose and manage IBD, evaluate extraintestinal manifestations, guide biologic therapy
Rheumatology	Assess axial and peripheral arthritis, manage DMARDs, NSAIDs, and biologics
Dermatology	Identify cutaneous signs (e.g., pustulosis, acne, and hidradenitis suppurativa), distinguish from mimics
Radiology	Interpret CT, MRI, and scintigraphy; identify characteristic signs such as the “bull’s head” pattern
Pathology	Rule out infection or malignancy on bone biopsy, confirm sterile inflammation
Infectious disease	Exclude osteomyelitis, guide cessation of unnecessary antibiotic therapy

Recommendations for early recognition

Who to Suspect

Early recognition of SAPHO-IBD overlap requires vigilance in specific patient populations. Clinicians should suspect SAPHO in patients with established IBD who develop unexplained bone pain, particularly in the anterior chest wall, mandible, or spine [14,46]. Conversely, individuals presenting with recurrent sterile osteitis or SAPHO-like skeletal pain should be evaluated for underlying IBD, especially when gastrointestinal symptoms or a family history of IBD are present [10,73].

Diagnostic Clues

Cutaneous manifestations such as palmoplantar pustulosis, hidradenitis suppurativa, and nodulocystic acne frequently precede osteitis and represent important diagnostic clues. Their recognition as systemic indicators rather than isolated skin disease can shorten diagnostic latency. Musculoskeletal involvement most often centers on the anterior chest wall, affecting the sternoclavicular, manubriosternal, and costochondral joints in more than 70% of reported cases [4]. Less typical sites such as the mandible and skull base have also been described, including the atypical case reported by Kim et al. [6].

Key Imaging

Magnetic resonance imaging (MRI) remains the most sensitive modality for detecting early osteitis and bone marrow edema. Whole-body scintigraphy can demonstrate multifocal or subclinical sterile osteitis, with the “bull’s head” sign regarded as pathognomonic in SAPHO [4,14]. Computed tomography (CT) is particularly useful for visualizing hyperostosis, erosions, and sclerosis, but may miss early inflammatory lesions.

Common Pitfalls

Diagnostic delays frequently result from misclassification as infectious osteomyelitis, leading to unnecessary prolonged antibiotic use, negative culture results, and non-diagnostic biopsies. Misdiagnosis as seronegative spondyloarthritis also occurs, particularly when sacroiliitis predominates and chest wall involvement is overlooked. Fragmented recognition of musculoskeletal, cutaneous, and gastrointestinal features in isolation further compounds delays [10].

Action Plan

Early multidisciplinary evaluation involving gastroenterology, rheumatology, and dermatology is essential when musculoskeletal, cutaneous, and gastrointestinal features coexist [74,75]. Such collaboration improves diagnostic accuracy, reduces unnecessary investigations, and informs the rational sequencing of biologic therapies. Treatment should be tailored to phenotype and biologic exposure; anti-TNF agents may provide dual remission but can provoke paradoxical dermatologic reactions, while ustekinumab and tofacitinib have shown promise in refractory cases [5,6,17]. Recurring clinical insights and their direct implications for practice are summarized in Table 4.

**Table 4:** Clinical pearls from the literature These case-based insights provide practical, real-world guidance that complements the broader diagnostic and therapeutic strategies outlined in earlier sections. SAPHO: synovitis, acne, pustulosis, hyperostosis, and osteitis, IBD: inflammatory bowel disease, GI: gastrointestinal, TNF: tumor necrosis factor

Insight	Clinical implication
Musculoskeletal symptoms often precede GI diagnosis.	Consider underlying IBD in patients with sterile osteitis or unexplained chest pain.
The anterior chest wall is the most commonly affected site.	Pain or swelling in this area should raise suspicion for SAPHO.
Cutaneous lesions may predate bone symptoms.	Dermatologic input is critical; avoid misdiagnosis as acne or psoriasis.
Anti-TNF agents show mixed efficacy.	Monitor for paradoxical flares; consider switching biologics if skin toxicity develops.
Ustekinumab and tofacitinib show promise post-TNF.	These agents offer dual control of intestinal and joint inflammation.
Misdiagnosis as an infection is common.	Avoid prolonged antibiotics or unnecessary biopsies if cultures remain persistently sterile.

Future directions

Need for Multicenter Collaboration

Despite growing awareness of SAPHO syndrome, its clinical overlap with inflammatory bowel disease (IBD) remains underrecognized, understudied, and largely absent from formal diagnostic or therapeutic guidelines. Current management approaches are extrapolated from related autoinflammatory and spondyloarthritic conditions, with very limited evidence addressing the specific SAPHO-IBD phenotype. Addressing these knowledge gaps will require multicenter collaboration, prospective data collection, and biomarker discovery. To achieve this, coordinated leadership from gastroenterology, rheumatology, and dermatology societies will be essential. These specialties are best positioned to develop multicenter registries that systematically capture symptom chronology, imaging findings, treatment exposures, and patient-reported outcomes.

Lack of Prospective Data

One major limitation is the lack of prospective data. The available evidence base consists almost entirely of case reports and small retrospective series, with no prospective cohorts or controlled trials systematically assessing disease course, treatment response, or relapse risk. A collaborative registry that tracks patients with IBD with extraintestinal sterile osteitis, hyperostosis, or anterior chest wall lesions could provide the foundation for predictive modeling, biomarker validation, and evidence-based recommendations.

Need for Consensus-Based Diagnostic Criteria

Diagnostic uncertainty also persists because no universally accepted criteria exist for SAPHO syndrome, and this is particularly problematic in the context of IBD. An actionable next step would be the creation of consensus-based diagnostic criteria developed jointly by gastroenterologists, rheumatologists, dermatologists, and radiologists, incorporating characteristic imaging signs (such as the “bull’s head” sign on scintigraphy and bone marrow edema without abscess on MRI) and explicit exclusion of mimicking conditions.

Development of Predictive Biomarkers

Another unmet need lies in the absence of predictive biomarkers. Conventional markers such as C-reactive protein (CRP) and erythrocyte sedimentation rate (ESR) are often normal even during active disease, and HLA-B27 expression is inconsistent. Future studies should prioritize validation of accessible biomarkers such as fecal calprotectin, serum S100 proteins, and IL-23 or IL-1β levels, while research consortia explore high-throughput tools (transcriptomics, proteomics, and microbiome profiling). Clinically, such biomarkers could help predict which patients with IBD are at risk of developing sterile osteitis, or which patients with SAPHO might respond best to TNF versus JAK inhibition.

Therapeutic Trials and Registries

Therapeutic uncertainty further complicates management, as no pharmacologic agent has been specifically approved for SAPHO syndrome. Treatment relies heavily on off-label use of biologics such as anti-TNF agents, ustekinumab, or tofacitinib, with responses varying across intestinal, cutaneous, and musculoskeletal domains. To generate actionable evidence, adaptive platform trials and therapeutic registries that evaluate outcomes across multiple disease domains should be prioritized. Such initiatives would inform cross-specialty treatment pathways and consensus algorithms.

Educational Initiatives

Educational gaps also hinder timely diagnosis. Practical measures could include incorporating SAPHO-IBD case vignettes into gastroenterology and rheumatology board curricula, using electronic decision support prompts for unexplained chest wall pain in patients with IBD, and expanding multidisciplinary case conferences that include dermatology and radiology. These strategies would reduce fragmented care and accelerate appropriate referral.

Pathophysiologic Exploration: The Gut-Bone-Skin Axis

From a pathophysiologic perspective, emerging evidence highlights the immunologic interdependence of the intestinal, skeletal, and dermatologic systems. The “gut-bone-skin axis” refers to how gut microbiome disruption and epithelial barrier dysfunction can trigger systemic inflammation that manifests as sterile osteitis and pustular skin lesions. Tissue-level immune profiling and autoantibody panels, although technical, could translate clinically by identifying at-risk patients before symptoms become multisystemic.

In summary, advancing the field will require a coordinated strategy that spans prospective registries, consensus-based diagnostic criteria, biomarker validation, therapeutic trials, and clinician education. These practical steps would transform SAPHO-IBD overlap from an underrecognized phenomenon into a well-characterized clinical entity with clear diagnostic standards and targeted management strategies.

## Conclusions

The coexistence of SAPHO syndrome and inflammatory bowel disease (IBD) presents a rare but clinically significant challenge, often leading to delays in diagnosis due to the asynchronous development of musculoskeletal, dermatologic, and gastrointestinal symptoms. Key features such as sterile osteitis, hyperostosis, and pustular skin lesions frequently mimic other conditions, complicating diagnosis, especially in patients with pre-existing IBD.

Although evidence is largely based on case reports, shared immunologic pathways, such as TNF-α, IL-23/Th17, and JAK-STAT signaling, have expanded treatment options. Targeted biologics such as infliximab, ustekinumab, and tofacitinib have shown promise, although responses vary, and paradoxical skin reactions remain a concern. Advanced imaging and bone biopsy are crucial for identifying subclinical lesions and ruling out other conditions. Bisphosphonates offer a non-immunosuppressive alternative in refractory cases. Early multidisciplinary collaboration is vital to improve diagnosis and optimize treatment. Future efforts should focus on creating consensus diagnostic criteria and individualized treatment algorithms to enhance patient outcomes.
